# Acute and Subchronic Toxicity Assessment of Conventional Soxhlet *Cymbopogon citratus* Leaves Extracts in Sprague–Dawley Rats

**DOI:** 10.1155/2023/8575741

**Published:** 2023-12-11

**Authors:** Jacob Apibilla Ayembilla, Abdul Raouf Khalid, Sharif Buari Abubakari, Abdul Rashid Adams, Felix Abekah Botchway, Stephen Antwi, Phyllis Naa Yarley Otu, Michael Appiah, George Osei-Adjei, Kwame Owen Kottoh, Peace Ahiabenu-Williams, Olga Quasie

**Affiliations:** ^1^Department of Science Laboratory Technology, Faculty of Applied Sciences, Accra Technical University, Accra, Ghana; ^2^Department of Medical Laboratory Technology, Faculty of Applied Sciences, Accra Technical University, Accra, Ghana; ^3^Department of Pharmacology & Toxicology, Centre for Plant Medicine Research, Mampong-Akuapem, Ghana; ^4^Department of Medical Laboratory Science, School of Biomedical and Allied Health Sciences, University of Ghana, Legon, Accra, Ghana

## Abstract

**Background:**

In Ghana, *Cymbopogon citratus* leaves together with guava, pawpaw, and lime are processed into a decoction to treat fever. To encourage its usage, preclinical validation of the safety profile of the plant is required. The acute and subchronic toxicities of the conventional Soxhlet ethanolic *Cymbopogon citratus* leaves extract in Sprague–Dawley rats were investigated.

**Methods:**

Pulverized *Cymbopogon citratus* leaves were extracted with 98% ethanol using the conventional Soxhlet extraction (CSE) method and dried. In the acute toxicity study, a single dose of 5000 mg/kg body weight was administered to six female Sprague–Dawley rats and 1 ml/100 g body weight normal saline to control (6) once, and signs of toxicity were observed every hour for the first 12 hr, 24 hr, and 48 hr through to 14 days. In the subchronic study, the treatment groups were administered 200 mg/kg, 600 mg/kg, and 1200 mg/kg, respectively, of the CSE *C. citratus* leaves extract for six weeks. Analyses were conducted on the blood, urine, and serum samples of the rats. Histopathological examination of the liver, heart, kidney, spleen, and lungs was carried out at termination. Analysis of variance (ANOVA) was performed to determine statistically significant differences between the test and control rats at *P*  <  0.05.

**Results:**

The results revealed that there were no statistically significant differences (*p*  >  0.05) in the urinalysis and haematological analysis between control and test rats over the treatment period. Similarly, CSE *C. citratus* leaves extract did not induce any significant biochemical changes in the treatment group; however, there was a weight loss effect on the treated rats. There were no noticeable morphological changes in the heart, liver, spleen, lung, and kidney of the test rats compared to the control.

**Conclusion:**

CSE ethanolic *C. citratus* leaves extract has a weight loss effect, and long-term administration of the extract may not cause any organ-specific toxicity to the consumers.

## 1. Introduction

Nowadays, pharmaceutical companies are extensively involved in the use of medicinal plants as antiparasitic, insecticidal, cosmetic, bactericidal, and fungicidal agents due to the nontoxic nature of secondary metabolites from such plants [1, 2]. Herbal medicines are alternatives to orthodox drugs due to their perceived low toxic effect on biological systems, low cost, and availability. It is believed that even if the expected therapeutic efficacy is not realized, their consumption is not dangerous. However, safety issues of herbal formulations have been raised by many due to poor scientific validation of the safety profile of these herbal medicines and reports of illness and death from their consumption [3–5]. Hepatotoxicity and nephrotoxicity are commonly reported toxic effects of herbal medicines due to the involvement of the liver and kidney in the metabolism and excretion of drugs [5, 6]. Thus, a comprehensive scientific study of the toxicity of these herbal products will provide the needed scientific evidence to support the argument that consumption of herbal concoctions is safe in humans. These studies are important for public health reasons to protect the user population from possible adverse side effects. There is a wide biodiversity of plant species with medicinal properties including *Cymbopogon citratus.*

*Cymbopogon citratus* with the common name lemongrass is a tufted, aromatic perennial grass with numerous stiff stems arising from a common rhizome. It is a tropical plant cultivated in South Asia, South and Central America, and Africa [7, 8] and used as an infusion or decoction in traditional medicine [8, 9]. *C. citratus* contains essential oils rich in ketones, alcohols, esters, terpenes, and aldehydes which vary according to the geographical origin of the plant [10]. It is reported to have phenolic and flavonoid, myrcene, and citral compounds. These compounds, depending on the concentration or dosage used, could be toxic to the user. For example, a study by Nogueira et al. [11] revealed that citral at 60 mg/kg bw.t induced maternal and embryofoeto-toxicity in rats. Also, citral induced cytotoxicity and genotoxicity in NCTC 929 mouse fibroblast cell lines [12, 13]. The LD_50_ of lemongrass essential oils against *Stitophilus granaries* is 6.92 *μ*g/insect [14]. Plants naturally produce metabolites as defensive agents against predators in their ecosystem which have the potential to harm rats, humans, and the environment at large [15].

In Brazil, *C. citratus* is used to alleviate mental disorders and gastrointestinal issues [16, 17]. It is said to have anxiolytic, anticonvulsant, and sedative effects [16]. It is an herbal medicine that is used in Thailand and Cuba to treat hypertension [18]. This plant is used to cure diabetes, obesity, and coronary heart disease in the eastern part of Nigeria [19]. *C. citratus* has antifungal, antimalarial, antibacterial, antidiarrhoeal, antifilarial, anti-inflammatory, deodorizing agents, antimutagenicity, antimycobacterial activity, antioxidant, hypoglycaemic, and antihyperlipidaemic activities [15–19].

In Ghana, *C. citratus* leaves are used in a decoction along with guava, pawpaw, and lime leaves to treat fever and are generally regarded as safe. To encourage its continuous usage, however, preclinical validation of the plant dose is necessary because the lethal dose (LD_50_) of the plant is unknown. Additionally, where the plant grows and how it is cultivated, harvested, and processed may change the chemical composition of the plant. Therefore, the purpose of this investigation was to ascertain the safety profile of *C. citratus* conventional Soxhlet ethanolic extracts when administered to Sprague–Dawley rats on an acute and subchronic basis.

## 2. Materials and Methods

### 2.1. Materials and Reagents

Automated Haematology analyzer (Mindray, China), Chemistry Analyzer (Mindray, China), total cholesterol (TChol), triglyceride (TRG), high-density lipoprotein cholesterol (HDL-c), low-density lipoprotein cholesterol (LDL-c), alanine aminotransferase (ALT), aspartate aminotransferase (AST), alkaline phosphatase (ALP), gamma-glutamyl transferase (GGT), bilirubin (total and direct), urea, creatinine, sodium, potassium, chloride reagents, diluent, and lyse all from Shenzhen Mindray Bio-Medical Electronics Co. Ltd., China, 80-2C centrifuge, urine strips (Urit Medical Electronics Co. Ltd., China), ethylene diamine tetra acetic acid (EDTA) tubes, and serum gel separator tubes (Greenlife, Canada), Sprague–Dawley rats were purchased from Centre for Plant Medicine Research (CPMR), Mampong Akwapim, Ghana. All reagents were analytical grade and solutions freshly prepared.

### 2.2. Study Setting

The extraction was carried out at the Department of Science Laboratory Technology of Accra Technical University, Ghana and the animal experimentation study was conducted at the Centre for Plant Medicine Research, Akwapim Mampong, Ghana, between August, 2022 and February, 2023.

### 2.3. Plant Material

Fresh leaves of matured *C. citratus* were harvested at Taifa Burkina, in the Ga East Municipal Assembly in the Greater Accra Region of Ghana in July 2022 and authenticated by a Botanist at the Plant Development Department at the Centre for Plant Medicine Research (CPMR). A voucher specimen of CPMR5167 was kept at the herbarium. The *C. citratus* leaves were cut into pieces, washed with distilled water, allowed to air dry for three weeks at room temperature, and pulverized into powder. The powder obtained was bagged into plastic zip lock bags and stored at room temperature until extraction. The extraction of *C. citratus* crude extracts was carried out by the conventional Soxhlet extraction (CSE) method as described as follows.

### 2.4. Conventional Soxhlet Extraction (CSE) of *C. citratus* Ethanolic Extract

The CSE technique was carried out according to the method described in [20, 21] with slight modifications. The extraction thimble was filled with a 15 g quantity of pulverized *C. citratus* powder, and the filled thimble was placed within the Soxhlet apparatus. A 250 ml of 98% ethanol was measured into a round bottom flask and connected to the extractor. Each cycle of extraction was performed for 3 hours. The temperature of the extraction corresponded to the boiling point of ethanol. After the CSE was completed, a rotary evaporator (RE-52A, E. Track Scientific Instruments, England) was used to concentrate the crude extract. The concentrate was transferred into a stainless plate and dried in a water bath at 70°C.

### 2.5. Ethical Considerations

The study was approved by the Research and Ethical Review Committee, Faculty of Applied Sciences, Accra Technical University (ATU/MLT/ET/01190301B/01190030B/2021-2022). Efforts were made to minimize suffering and reduce the number of animals used by regular provision of food and water, cleaning out the bedding of the animals every other day, allowing the rats to sleep in a calm and dark environment at night, and monitoring them throughout the study period for any discomfort or distress. The study was conducted according to internationally accepted standards and principles of laboratory animal use and care (EEC directive of 1986:86/609/EEC) [22].

### 2.6. Sample Size Calculation

The sample size for the study was calculated using the resource equation approach and analysis of variance (ANOVA) as the expected statistical analysis [23, 24].

(1) $$\begin{array}{c} n=\frac{DF}{k}+1, \end{array}$$

where DF = degrees of freedom (range between 10 and 20), *k* = number of groups, and *n* = number of rats per group. For the four groups used for this study, a sample size of range between 4 and 6 rats per group was calculated. Thus, six rats per group were used for the study.

### 2.7. Experimental Rats

Sixty days old healthy female (nulliparous and nonpregnant) Sprague–Dawley rats (125−219 g) were obtained from the Animal Experimental Unit, Department of Pharmacology & Toxicology of the Centre for Plant Medicine Research (CPMR), Mampong-Akuapem, in the Eastern Region of Ghana. The selection of female rats was based on the OECD guideline tests No. 423 and No. 408 primarily due to the better sensitivity of female rats than males [25, 26]. The rats were maintained at a room temperature of 22 ± 2°C and humidity of 55 ± 10% with a 12 h light/dark cycle [27]. They were allowed access to rodents' pelleted feed (crude protein 17.09%, ether extract 3.368%, crude fibre 3.35%, calcium 3.0%, phosphorus 0.56%, lysine 0.76%, methionine 0.46%, and metabolizable energy 2,873.84 kcal/kg) purchased from Agricare Limited, Kumasi, Ghana, and sterilized distilled water *ad libitum*.

### 2.8. Acute Toxicity Study

The acute toxicity study was conducted according to the OECD guidelines tests No. 420 and 423 [25]. Female (nulliparous and nonpregnant) Sprague–Dawley rats were randomly grouped into two groups (*n* = 6) and allowed to acclimatize for a week. Both groups fasted overnight but were allowed access to distilled water ad libitum the night before the day of the experiment. Group 1 (treatment) was administered with 5000 mg/kg bw.t single dose of the extract orally using oral gavage. Group 2 (control) was administered orally 1 ml/100g bw.t normal saline. The rats were then observed every hour for the next 12 hours and then at 24 hr, 48 hr till the fourteenth day for general changes in behavioural and physiological function as well as mortality. The assessments were carried out according to the primary observation procedure by the Irwin test [28].

### 2.9. Subchronic Toxicity Study

The subchronic toxicity study was conducted according to the Organization of Economic Cooperation and Development (OECD) guidelines 407 [29]. The protocols used in [27, 30] were used with slight modifications.

Sixty-day-old female (nulliparous and nonpregnant) Sprague–Dawley rats were randomly divided into four groups (*n* = 6) and allowed to acclimatize for one week. They were fed with rat chow and sterilized distilled water *ad libitum*. The rats were grouped into the following treatment groups, respectively:

  Group I: negative control (sterilized distilled water)
  Group II: low dose (200 mg/kg bw.t)
  Group III: medium dose (600 mg/kg bw.t)
  Group IV: high dose (1200 mg/kg bw.t)

The extracts were orally administered to the rats daily for six weeks (42 days). The rats were monitored closely daily for any signs of toxicity. Appearance and behavioural changes were also assessed, and any abnormalities in water and food intake were recorded according to the primary observation procedure of the Irwin test [28]. The rats were weighed at baseline, 2^nd^, 3^rd^, 4^th^, 5^th^, and 6^th^ weeks. Blood and urine samples were collected for analysis at baseline, 3^rd^, and 6^th^ weeks. The liver, kidney, heart, spleen, and lungs were excised at termination for histopathological examination.

### 2.10. Urinalysis

Fresh, clean urine samples for the analysis were collected by holding each rat over a tabletop and manually expressing its bladder by application of gentle transabdominal pressure and urine delivered onto the tabletop [31]. A urine dipstick was immersed into the urine, and the strips were bloated with soft tissue to prevent cross-contamination. The colour changes on the strips were compared with the urine colour chart. The urinalysis was done at baseline, 3^rd^, and 6^th^ weeks for glucose, proteins, ketones, leucocytes, nitrite, bilirubin, specific gravity, pH, urobilinogen, and blood using urine biochemical strips (Urit Medical Electronics Co. Ltd., China).

### 2.11. Blood Sampling and Isolation of Organs

Blood samples of the rats in each treatment group were obtained by tail strains (at baseline, 3^rd^, and 6^th^ weeks) into EDTA anticoagulated tubes for haematological analysis and into serum gel separator tubes (SSTs) for serum biochemical analysis. Blood in the EDTA tubes was gently swirled and inverted about 5 to 10 times to ensure EDTA was well mixed with the blood to prevent clotting. Blood in the SST was allowed to clot for 20 minutes and centrifuged at 5000 g for 5 min. The serum obtained was stored at −20°C until analysis.

At termination, the rats in both the treatment and control groups were sacrificed by cervical dislocation and dissected. The heart, liver, kidney, spleen, and lungs were excised, freed of fat and connective tissues, bloated on clean tissue paper, and weighed on an electronic balance. The tissues were fixed in 10% neutral buffered formalin (pH=7.2) for histopathological examination [32].

### 2.12. Effect of CSE *C. citratus* Ethanolic Extract on Haematological Parameters

Full blood count (FBC) of the rats was performed using a fully automated haematology analyzer (Shenzhen Mindray Bio-Medical Electronics Co., Ltd., China). The measured parameters were white blood cells (WBCs), lymphocytes (lymph#), granulocytes (Gran#), red blood cells (RBCs), haemoglobin (Hb), haematocrit (HCT), mean corpuscular volume (MCV), mean corpuscular haemoglobin (MCH), mean corpuscular haemoglobin concentration (MCHC), red cell distribution width (RDW), platelet (PLT), platelet distribution wide (PDW), mean platelet volume (MPV), and platelet crit (PCT).

### 2.13. Effect of CSE *C. citratus* Ethanolic Extract on Serum Biochemical Parameters

Serum biochemical analysis was performed on serum to determine the effect of the extracts on lipid metabolism, liver, and kidney function. The following parameters were measured for lipid profile: total cholesterol (TChol), triglyceride (TRG), high-density lipoprotein (HDL-C), low-density lipoprotein (LDL-C), and coronary risk (CR) was calculated by dividing the total cholesterol by HDL-C; for liver function tests: total protein (TP), albumin (ALB), alkaline phosphatase (ALP), alanine aminotransferase (ALT), aspartate aminotransferase (AST), gamma-glutamyl transferase (GGT), total bilirubin (BIT), and direct bilirubin (BID); for kidney function: urea, creatinine (Crea), sodium (Na^+^), potassium (K^+^), and chloride (Cl^−^) using a fully automated chemistry analyzer (Shenzhen Mindray Bio-Medical Electronics Co. Ltd., China).

### 2.14. Effect of CSE *C. citratus* Ethanolic Extract on Body and Organ Weights of Rats

The body weights of the rats were recorded at baseline, 3^rd^, and 6^th^ weeks. The percentage change in body weight of the rats was calculated as follows:

(2) $$\begin{array}{c} \text{Percentage body weight gain}=\frac{\text{Weekly weight }\left(g\right)\;-\text{Baseline weight}\left(g\right)}{\text{Baseline Body Weight }\left(g\right)}\;x\;100\%. \end{array}$$

The relative organ weight (ROW) of each organ was calculated as follows:

(3) $$\begin{array}{c} \text{Relative Organ Weight}=\frac{\text{Absolute Organ Weight }\left(g\right)}{\text{Rat Body Weight }\left(g\right)}\;x\;100\%. \end{array}$$

### 2.15. Histopathological Examination

Portions of the organs were excised and processed into paraffin blocks in labeled tissue processing cassettes. Each was passed through ascending grades of alcohol (70%, 80%, 90%, and absolute). They were further dehydrated with two changes of absolute alcohol cleared in three changes of xylene and ultimately infiltrated and embedded in paraffin wax. Sections of 4 µm thickness were cut from each block, mounted on a microscope slide, and stained with haematoxylin and eosin stain [32]. The stained tissues were observed with an Olympus microscope for morphological changes and photographed.

### 2.16. Statistical Analysis

The data were entered into Microsoft Excel 365 for cleaning, analysis, and plotting of graphs and tables. The results obtained were reported as mean ± standard error of the mean (SEM). The data were further exported into GraphPad Prism version 8.4.2 (GraphPad Software, San Diego, CA, USA) for one-way analysis of variance (ANOVA) analysis. The ANOVA was used to determine whether there was a statistically significant difference between the control and experimental groups followed by Tukey's multiple comparisons test. *P*  <  0.05 was considered statistically significant.

## 3. Results

### 3.1. Acute Toxicity Study

All the rats were observed up to 12, 24, and 48 hr, 7 days, and 14 days after the treatment. No mortality was recorded (Table 1). No physical evidence of toxicity such as piloerection, diarrhoea, increased urination, salivation, lachrymatory, locomotory defects, difficulty in breathing, or asthenia was recorded (Table 1). This, therefore, suggests that the oral median lethal dose (LD_50_) of CSE *C. citratus* ethanolic extract is greater than 5000 mg/kg.

### 3.2. Subchronic Toxicity Study

#### 3.2.1. Percentage Body Weight Gain

**Figure 1:**
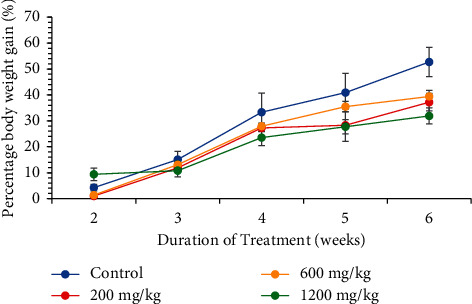
Effect of oral administration of CSE *C. citratus* ethanolic extract on the percentage body weight of the rats over a six-week treatment period. Each data point represents mean ± SEM (*n* = 6).

**Figure 2:**
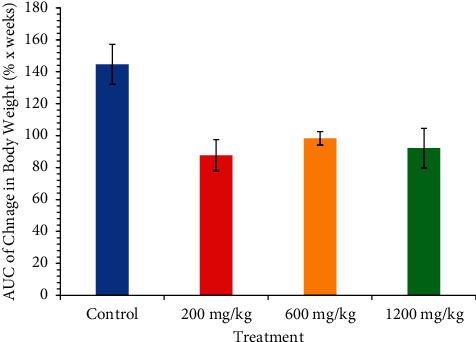
Area under the curve analysis of percentage body weight gain over the six-week treatment period. Each bar represents the mean total AUC ± SEM of each treatment group line graph in Figure 1. AUC: area under the curve.

The effect of CSE *C. citratus* ethanolic extract on the weekly percentage body weight gain of the rats is shown as follows (Figure 1). Both the control and the test rats gained weight steadily over the study period. However, the control rats gained more weight than the test rats, which was not statistically significant, *p*  >  0.05. The rats in the 600 mg/kg bw.t and 1200 mg/kg bw.t treatment groups gained comparatively the same amount of weight, which was higher than the weight gained by the 200 mg/kg, but this was not statistically significant, *p*  >  0.05. The area under the curve (AUC) analysis confirms this trend of growth of the rats (Figure 2). The control group had the highest AUC (144.6, 95% CI [104.3, 184.9]) followed by the 600 mg/kg bw.t group (98.23, 95% CI [89.90, 106.6]). The 200 mg/kg bw.t and the 1200 mg/kg bw.t had comparatively the same AUC (87.58, 95% CI [68.11, 107.0]) and (92.06, 95% CI [44.01, 140.1]), respectively, though the uncertainty in the 1200 mg/kg bw.t group is higher because of its wider confidence interval. The AUC was not statistically significantly different between and among the groups (*p*  >  0.05) (Figure 2).

#### 3.2.2. Effect of CSE *C. citratus* Ethanolic Extract on Lipid Profile

Table 2 shows the effect of the CSE ethanolic *C. citratus* extract on the lipid profile of the control and test rats at the termination of the experiment. The total cholesterol and HDL-c were higher in the control than in the test rats, and for the treatment groups, the total cholesterol was higher in all test rats than HDL-c. However, triglycerides and LDL-c were higher in the test rats than in the control rats. The triglyceride concentration was in order 600 mg/kg > 1200 mg/kg > 200 mg/kg > control group. However, for LDL-c, the 200 mg/kg bw.t treatment group recorded the highest, followed by 1200 mg/kg bw.t and then 600 mg/kg bw.t with the control group being the least. The coronary risk (CR) was higher in the 1200 mg/kg bw.t treatment followed by the control group, followed by 600 mg/kg bw.t and 200 mg/kg bw.t being the least. No statistically significant difference was found at *p*  <  0.05 for all parameters measured between the control and test rats and within the test rats.

#### 3.2.3. Percentage Organ to Body Weight Ratio

**Figure 3:**
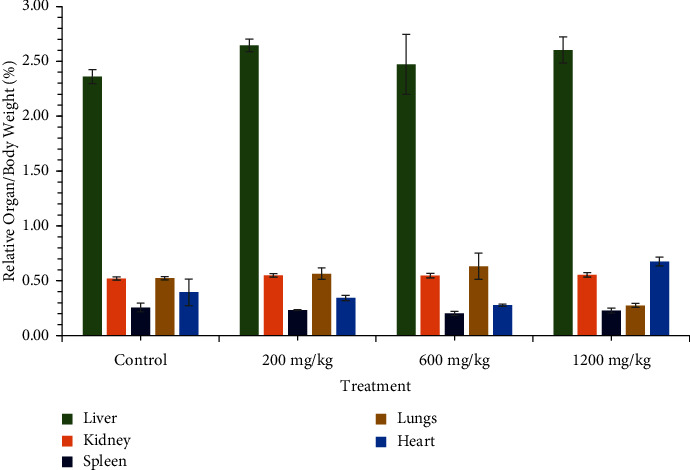
Effect of oral administration of CSE *C. citratus* ethanolic extract on percentage organ/weight at the termination of the experiment. Each bar represents mean ± SEM (*n* = 6), (*p*  >  0.05).

The effect of the CSE ethanolic extract on the relative organ/body weight ratio (%) at the termination of the experiment of the control and test rats is plotted in Figure 3. The results showed no significant changes (*p*  >  0.05) in the organ weights expressed as a percentage of body weight between the control and test rats for each treatment group. The relative organ/body weight ratio (%) of the lungs decreased at 1200 mg/kg bw.t compared with the other treatment groups and control rats in the CSE treatment group. However, that of the heart increased in the 1200 mg/kg treatment compared to the control and other treatment groups in the CSE treatment group. These differences were however not statistically significant (*p*  >  0.05).

#### 3.2.4. Effect of CSE *C. citratus* Ethanolic Extract on Haematological Parameters

Table 3 shows the effect of CSE ethanolic *C. citratus* extracts on haematological indices of Sprague–Dawley rats at the termination of treatment. The results show that there were no statistically significant differences in all parameters measured between the control and test rats (*p*  >  0.05). The WBC (18.6 × 10^9^)/L, Lymph# (11.98 × 10^9^)/L, Mid# (1.50 × 10^9^)/L, and Lymph% (68.05%) of the control group were higher than WBC (10.15–13.18)10^9^/L, Lymph# (6.58–6.85)10^9^/L, Mid# (0.88–1.13)10^9^/L, and Lymph% (58.73–64.68%) of the test rats. Gran% (23.58%) of the control rats was lower than the test rats' Gran% (26.55–32.48).

#### 3.2.5. Effect of CSE *C. citratus* Ethanolic Extract on Serum Biochemical Parameters

*(1) Liver Function Test*. The effect of subchronic oral administration of CSE extracts on rats' serum liver function test is shown in Table 4. The activities of serum alanine aminotransferase (ALT), aspartate aminotransferase (AST), alkaline phosphatase (ALP), and *γ*-glutamyl transferase (GGT) were determined. The serum ALT activity was relatively the same for the control, 600 mg/kg bw.t, and 1200 mg/kg bw.t treatment groups. However, there was an increase in its activity in the 200 mg/kg bw.t treatment group than the control, 600 mg/kg bw.t, and 1200 mg/kg bw.t. However, for AST, its activity was relatively the same among the control and 600 mg/kg bw.t treatment groups. Moreover, the 200 mg/kg w.t and 1200 mg/kg bw.t was almost the same, which was lower than the control and 600 mg/kg bw.t. In the case of ALP, the activity of the enzyme was higher in the treatment group than the control with 200 mg/kg bw.t having the highest activity following 120 mg/kg bw.t and 600 mg/kg bw.t being the least. For GGT, on the other hand, the activity of the enzymes was almost the same for the control and test groups. There was no statistically significant difference (*p*  >  0.05).

There was no statistically significant difference between the control and test rats' total protein, albumin, and bilirubin concentrations. The total protein was comparatively the same among the test groups and controls although concentration was decreased in this order: control >200 mg/kg > 1200 mg/kg > 200 mg/kg which was not statistically significant. For albumin, the concentrations were almost the same between control and test rats (Table 4). Total bilirubin was higher followed by indirect bilirubin and direct bilirubin was the least. The direct bilirubin was comparable between control and test rats. However, total bilirubin concentration increased as the dosage of treatment increased (200 mg/kg < 600 mg/kg < 1200 mg/kg) in the treatment groups. Total bilirubin in the control rats was higher than that in the 200 mg/kg treatment group but less than in the 600 mg/kg and 1200 mg/kg treatment group. The same trend was observed for indirect bilirubin. There was, however, no statistically significant difference (*p*  >  0.05) (Table 4).

*(2) Renal Function Test*. The serum concentrations of urea, creatinine, and electrolytes were measured after six weeks of oral administration of CSE extracts to control and test rats. The serum urea concentration was comparatively the same among the control and test rats (Table 5). The creatinine concentration of the 600 mg/kg bw.t treatment was the highest, followed by 1200 mg/kg bw.t, and then the control rats with the 200 mg/kg bw.t treatment were the least which was not statistically significant. The serum electrolyte concentrations were relatively the same between the control and test rats, respectively. Serum sodium concentration was higher followed by chloride concentration and potassium concentration was the least between the control and test rats (Table 5).

### 3.3. Urinalysis

Dipstick urinalysis data at the termination of the experiment following six weeks of oral administration of CSE *C. citratus* ethanolic extract is shown in Table 6. The results indicate that there were no significant differences in the levels of urine parameters between the control and test rats.

### 3.4. Histopathological Examination

**Figure 4:**
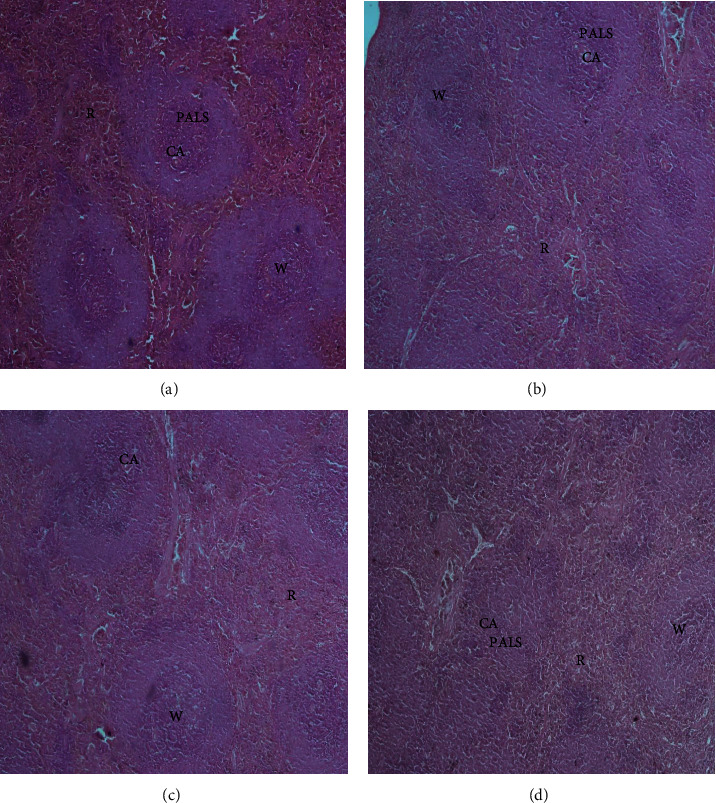
Representative micrographs of the spleen tissues in Sprague–Dawley rats after six weeks of CSE *C. citratus* ethanolic extract administration (x40). The micrographs show the white pulp (W) composed of lymphoid tissue with periarteriolar lymphoid sheaths (PALS) surrounding central arterioles (CA). Red pulp (R) regions show splenic cords composed of reticular fibers, macrophages, venus sinuses, and red blood cells. No significant inflammation, fibrosis, or abnormal cellularity was observed. (a) Spleen tissue of control rats treated with sterilized distilled water. (b) Spleen tissue of rats treated with 200 mg/kg bw.t CSE *C. citratus* ethanolic extract. (c) Spleen tissue of rats treated with 600 mg/kg bw.t CSE *C. citratus* ethanolic extract. (d) Spleen tissue of rats treated with 1200 mg/kg bw.t CSE *C. citratus* ethanolic extract.

**Figure 5:**
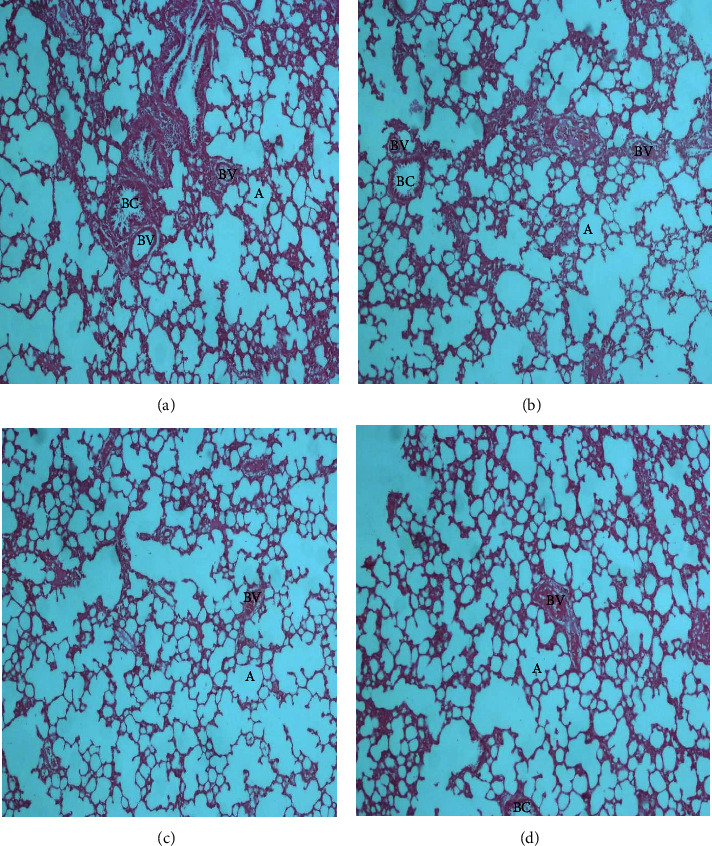
Representative micrographs of the lung tissue in Sprague–Dawley rats after six weeks of CSE *C. citratus* ethanolic extract administration (x100). The micrographs show normal lung architecture with alveolar spaces (A), bronchiole (BC), and blood vessels (BV) with no inflammation, fibrosis, or neoplastic change in all treatment groups. (a) Lung tissue of control rats treated with sterilized distilled, (b) lung tissue of rats treated with 200 mg/kg bw.t CSE *C. citratus* ethanolic extract, (c) lung tissue of rats treated with 600 mg/kg bw.t CSE *C. citratus* ethanolic extract, and (d) lung tissue of rats treated with 1200 mg/kg bw.t CSE *C. citratus* ethanolic extract.

**Figure 6:**
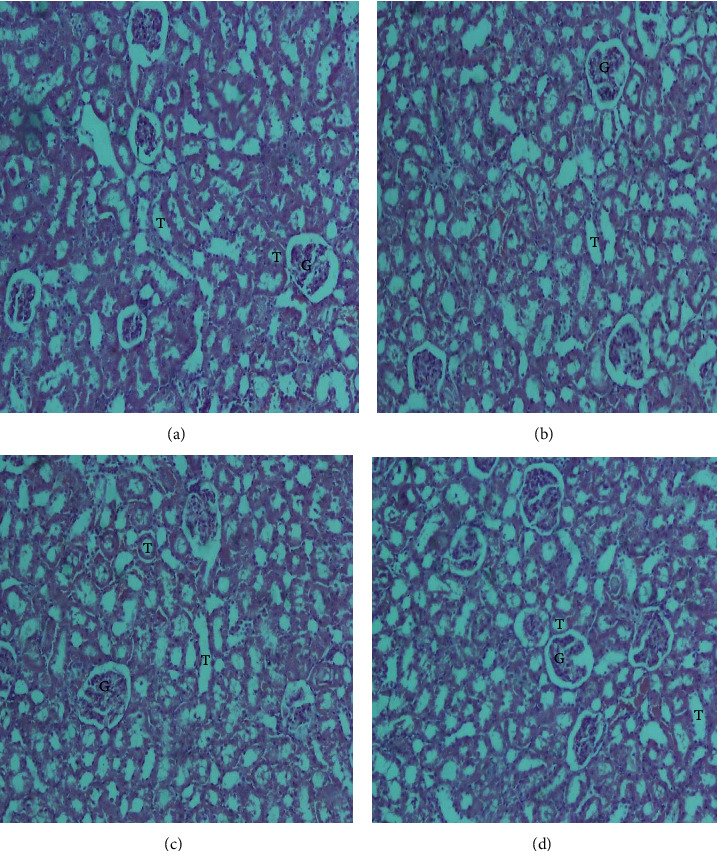
Representative micrographs of the kidney tissue in Sprague–Dawley rats after six weeks of CSE *C. citratus* ethanolic extract administration (x100). The micrographs show mainly glomeruli (G) and renal tubules (T) lined by a single layer of epithelial cells with regular nuclei. No tubular atrophy, interstitial fibrosis, or inflammatory infiltrates are noted. (a) Kidney tissue of control rats treated with sterilized distilled water. (b) Kidney tissue of rats treated with 200 mg/kg bw.t CSE *C. citratus* ethanolic extract. (c) Kidney tissue of rats treated with 600 mg/kg bw.t CSE *C. citratus* ethanolic extract. (d) Kidney tissue of rats treated with 1200 mg/kg bw.t CSE *C. citratus* ethanolic extract.

**Figure 7:**
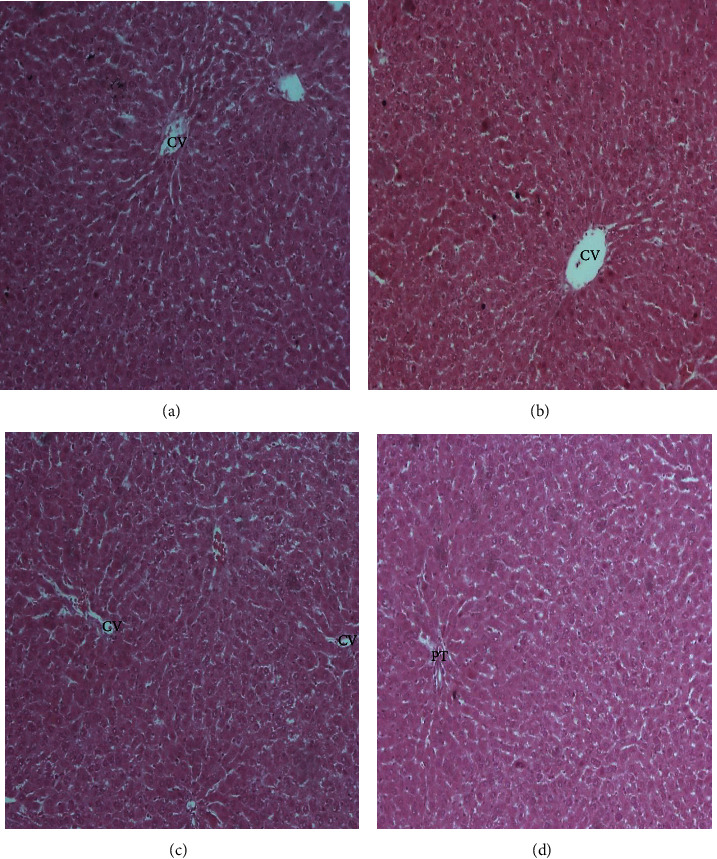
Representative micrographs of the liver tissue in Sprague–Dawley rats after six weeks of CSE *C. citratus* ethanolic extract administration (x100). The micrographs show central veins (CV) surrounded by radiating plates of hepatocytes. Also shows blood vessels, including portal triads (PT) consisting of a branch of the hepatic artery, portal vein, and bile duct. No inflammatory cells present and necrosis in all treatment groups. (a) Liver tissue of control rats treated with sterilized distilled water. (b) Liver tissue of rats treated with 200 mg/kg bw.t CSE *C. citratus* ethanolic extract. (c) Liver tissue of rats treated with 600 mg/kg bw.t CSE *C. citratus* ethanolic extract. (d) Liver tissue of rats treated with 1200 mg/kg bw.t CSE *C. citratus* ethanolic extract.

**Figure 8:**
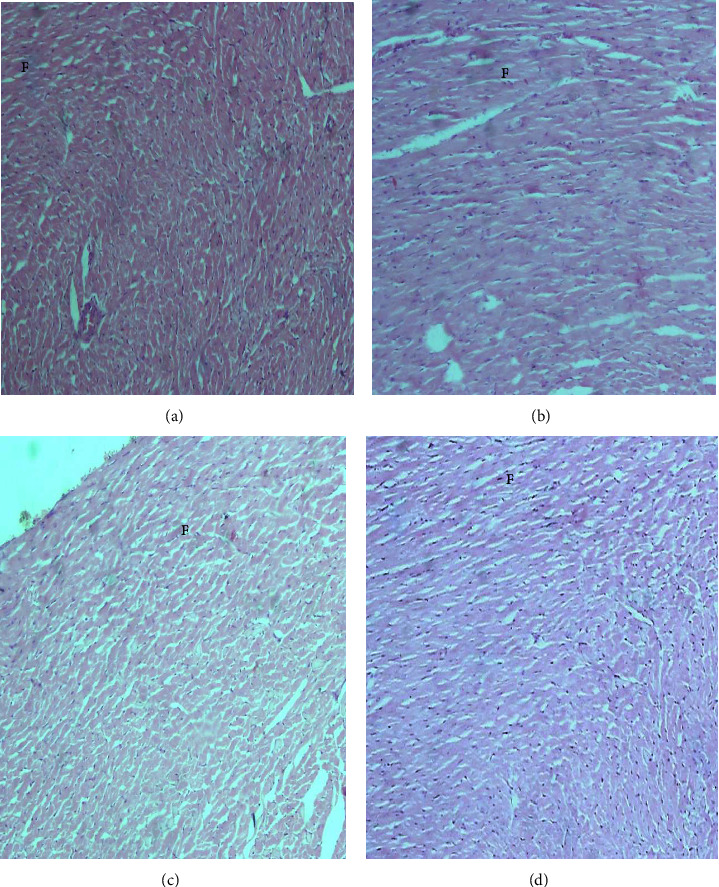
Representative micrographs of the heart tissue in Sprague–Dawley rats after six weeks of CSE *C. citratus* ethanolic extract administration (x100). Micrographs showing the characteristic branching arrangement of myocardial fibres (F) with centrally placed nuclei. No inflammatory cells are present and no myocardial infarcts. (a) Heart tissue of control rats treated with sterilized distilled water. (b) Heart tissue of rats treated with 200 mg/kg bw.t CSE *C. citratus* ethanolic extract. (c) Heart tissue of rats treated with 600 mg/kg bw.t CSE *C. citratus* ethanolic extract. (d) Heart tissue of rats treated with 1200 mg/kg bw.t CSE *C. citratus* ethanolic extract.

The histopathological examination of the organs isolated from the rats after six weeks of subchronic toxicity studies revealed that there was no abnormal histologic finding in sections of the liver, kidney, lung, heart, and spleen among the control and treatment groups (Figures 4–8). The cellular integrity and topologies were intact when comparing all tissues in the treatment groups to their respective controls.

## 4. Discussion

The increasing patronage of herbal preparations exposes consumers to toxicity due to poor scientific validation of the efficacy and toxicity of these herbal preparations, poor monitoring and regulation by regulatory authorities, and misidentification of the right species of plant for the treatment of a specific disease. *C. citratus* decoction is used in Ghana for the treatment of fever but the safety profile of the plant is not known. This study aimed to assess the safety profile of *C. citratus* extract by the Conventional Soxhlet ethanolic extraction method in Sprague–Dawley rats by acute and subchronic oral administration.

The acute toxicity study of the extract showed that a single dose oral administration of CSE ethanolic *C. citratus* extract to the rats did not produce any adverse effects (i.e., asthenia, defaecation, salivation, abnormal respiration, piloerection, locomotor, and lachrymatory activities) at a concentration of 5000 mg/kg bw.t. These results corroborate a report by Ayenew et al. [15], who reported that *C. citratus* extract showed no signs of toxicity and treatment-related mortality in rats up to 5000 mg/kg bw.t oral administration. It thus, suggests that the lethal dose of CSE ethanolic *C. citratus* extract is greater than 5000 mg/kg bw.t (LD_50_ > 5000 mg/kg bw.t).

The clinical relevance of acute toxicity studies is limited since bioaccumulation of toxic substances can cause severe adverse effects even at very low doses. Thus, multiple-dose administration is critical in ascertaining the true safety profile of drugs of interest [33]. Subchronic oral administration of the extracts was thus performed at dosages of 200 mg/kg bw.t, 600 mg/kg bw.t, and 1200 mg/kg bw.t, respectively. Treatment with CSE *C. citratus* ethanolic extract resulted in decreased weight gain in the test rats compared to the control (Figure 1). The weight loss effect of CSE *C. citratus* ethanolic extract could be due to appetite inhibiting or lipid-lowering effect. The findings reported here agree with [19]. The extract was shown to have cholesterol and HDL-c lowering effect (Table 2), but the hypothesis of appetite inhibiting effect requires validation.

Hypercholesterolaemia, obesity, and diabetes are closely linked to stroke and hypertension. Since the extract lowered the cholesterol levels in the test rats more than in the control, it lowered the cardiovascular risk of the test rats more than the control except for the 1200 mg/kg bw.t treatment group although LDL-c was higher in the test rats than in the control (Table 2). Thus, consumption of *C. citratus* extract has a low coronary risk. However, the borderline high triglyceride in the test rats than in the control suggests that continuous consumption of this extract could lead to pancreatitis and fatty liver disease due to triglyceride infiltration of these organs.

Relative organ-to-body weight ratio is a more sensitive marker of specific organ toxicity than absolute body weight, since the deleterious effect of a drug can be identified when a sensitive organ is affected [27, 32]. The results in this study revealed no significant change when the weight of the excised liver, heart, spleen, lungs, and kidney was expressed as a percentage relative to the mean body weight of the test rats. The lack of significant differences in the percentage relative organ weight to body weight affirms the safety profile of *C. citratus* CSE extracts (Figure 3).

The main transport system of drugs and xenobiotic products is the blood, and the cells of the blood interact with these substances during their stay and transport in the blood, making it one of the most vulnerable systems to pathological substances [34]. White blood cells are the first line of defense against infection, inflammation, or cellular injury [34]. Studies have reported that an increase in the level of WBC and its indices in test rats during the herbal extract subchronic administration results from the extract stimulating an immune response or inducing an inflammatory condition in the treated rats. However, a significant decrease in WBC count will signify a decrease in the production of leukocytes termed leukopenia, which reflects the reduced ability of the body to fight infection [35]. As recorded in Table 3, since the WBC and its indices of the test rats were normal and lower than that of the control following subchronic oral administration of *C. citratus* extract suggests that the long-term administration of CSE ethanolic *C. citratus* extract protected the test rats against infections and did not elicit an inflammatory response in the test rats. This finding explains why *C. citratus* decoction is used as a fever tea in Ghana. This study contradicts a study by Sadi and Imam [9], who reported that the WBC and its indices of test rats treated with aqueous and ethanolic *C. citratus* extracts were comparatively the same as the control. This study however agrees with Nosiri et al. [36], who recorded a decrease in WBC and its index in test rats compared to the control. A similar observation was made by Ekpenyong et al. [10], in healthy volunteers who after 30 days of receiving oral infusion of *C. citratus* infusion had significantly low WBC differential count. The RBC, Hb, HCT, MCV, MCH, and MCHC count in both control and test rats were comparatively the same. It implies that CSE *C. citratus* ethanolic extract has no harmful effect on bone marrow function. The normal count of the RBC indices (MCV, MCH, and MCHC) indicates that the RBCs were of normal morphology, and the extracts may be rich in vitamin B, folate, and Fe because a deficiency in these minerals would have manifested in structural abnormalities of the RBCs [8]. This study agrees with a study by Ayenew et al. [15], who observed that there was no alteration in RBC indices between control and test rats when administered with *C. martini* essential oil in mice. The platelet count (848–915)10^9^/L of the test rats of CSE *C. citratus* extract treatment groups was higher than that of the control (772 × 10^9^)/L which was not statistically significant, (*p*  >  0.05). The platelet-boosting effect of CSE extract suggests that *C. citratus* leaves may contain compounds with antioxidant activities such as vitamin C [8] which prevents platelet damage. *C. citratus* extract may thus have a thrombocytosis-promoting effect.

ALT and AST are intrahepatic enzymes that escape out of the hepatocytes to the blood following altered permeability of the hepatocellular membrane. They are transaminases involved in amino acid and carbohydrate metabolism. Elevation of the activities of these soluble cytosolic enzymes is a predictive marker of hepatocellular injury. The results from this study revealed that there were no significant differences between the activities of ALT and AST of the control and test rats (Table 4). This suggests that CSE ethanolic *C. citratus* extracts do not contain compounds that may have a membrane-damaging effect on the hepatocytes and thus adenosine triphosphate (ATP) synthesis, amino acids, and carbohydrate metabolism was not impaired [37, 38] in the test rats.

ALP and GGT are cholestatic-inducing enzymes of hepatobiliary origin with normal activity in normal hepatic function [39, 40]. They are employed to diagnose biliary flow obstruction or cholestasis. Of course, there are four isoforms of ALP with different tissues of origin (i.e., bone, placenta, intestinal, and liver). An increase in the activity of these isoforms could cause an increase in the activity of ALP. Moreover, for GGT, aside from cholestatic induction of increased GGT activity, drugs such as warfarin, alcohol, phenytoin, phenobarbital, and other anticonvulsant drugs induce the synthesis and increased activity of GGT [41, 42]. The absence of a nonsignificant increase in GGT activity suggests that CSE ethanolic extracts of *C. citratus* have no secondary metabolite with GGT induction activity. Since GGT activity was normal, the increase in the activity of ALP in the test rats to the control is not of liver origin. There were no statistically significant differences between the activities of ALP and GGT in the control and test rats (Table 4). This study agrees with the study [9] which also reported that *C. citratus* extract did not induce elevation of hepatic enzymes in albino rats. Indeed, reports in [43, 44] indicate that *C. Citratus* has a hepatoprotective effect by significantly decreasing the activity of hepatic enzymes compared to controls.

The concentrations of total protein and albumin were the same in both control and test rats as recorded in Table 4. The liver synthesizes all kinds of plasma proteins such as acute phase proteins involved in the eliciting of an inflammatory response, clotting factors, and intrinsic and extrinsic pathway factors [45]. A measurable decrease in total protein and albumin concentration reflects either impaired hepatocellular production, increased renal loss, or increased catabolism, which may occur in various pathological conditions [45]. The normal concentrations of total protein and albumin affirm the safety profile of *C. citratus* extract. The results indicate that consumption of *C. citratus* extracts does not impair the liver's synthetic function nor increase glomerular permeability to proteins.

Bilirubin is a product of the catalytic degradation of haemoglobin in red blood cells by haem oxygenase and biliverdin reductase [46]. Increased bilirubin synthesis occurs in haemolytic conditions like glucose 6-phosphate dehydrogenase deficiency (G6PD), sickle cell disease, ABO, and rhesus incompatibility, etc., resulting in anaemia [43, 44]. In this study, the total bilirubin for the control was higher than that for the 200 mg/kg bw.t treatment group, however, the total bilirubin for the 600 mg/kg bw.t and 1200 mg/kg bw.t treatment groups was higher than that for the control and 200 mg/kg bw.t treatment group. A similar trend was observed for indirect bilirubin (Table 4). This is suggestive that the increase in total and indirect bilirubin for 600 mg/kg and 1200 mg/kg bw.t is due to preanalytical errors but not pathological reasons because of the effect of the extract treated. Due to the tail strains method used, the RBCs could be easily haemolyzed, if the haemolysis was due to the extract, the control group should not have higher total and indirect bilirubin than the 200 mg/kg bw.t treatment. The result shows that CSE ethanolic extract of *C. citratus* did not induce haemolysis and for that matter anaemia in the test rats. The normal Hb levels measured in control and test rats in this study buttressed this point. Contrary to our report, Eraj et al. [43] reported a decreased level of bilirubin in test rats compared to controls when treated with *C. citratus* extract.

Serum urea, creatinine, and electrolytes are markers routinely measured to assess renal integrity. However, creatinine is a waste product of muscle metabolism and urea is a waste product of protein metabolism. The accumulation of these metabolites in the serum reflects renal impairment. The result from this study shows that urea concentrations were almost the same in the control and test rats (Table 5). This is in contrast with a study by Tarkang et al. [47], who reported elevation of serum urea following 28 days of administration of *C. citratus* ethanolic and aqueous extracts to Swiss albino rats. Creatinine concentrations were nonsignificantly higher in the 600 mg/kg and 1200 mg/kg treatment groups than in the control. These results suggest that *C. citratus* extract is not nephrotoxic. However, Ekpenyong et al. [8] reported that following 30 days of oral administration of *C. citratus* infusion, creatinine clearance (CCr), and estimated glomerulus filtration rate (eGFR) were decreased in healthy volunteers. They also observed a time- and dose-dependent decrease in CCr and eGFR, suggesting that prolonged and high-dose consumption of *C. citratus* extracts will be nephrotoxic to the consumer.

The measured electrolytes, sodium, chloride, and potassium in this study did not vary between control and test rats (Table 5). This suggests that *C. citratus* did not cause electrolyte derangement in the test rats. These findings agree with Christopher [48], who reported a nonelevation of serum electrolyte concentrations following 30 days of oral infusion of aqueous and ethanolic *C. citratus* extracts in healthy volunteers. They, however, reported a decrease in urinary and plasma pH at the end of the treatment compared to baseline and an increase in urinary excretion of electrolytes (i.e., Na^+^ and K^+^) suggesting that chronic consumption of *C. citratus* infusion could cause *C. citratus* associated acid-base derangement and electrolytes wastage. This report is contrary to our findings in this study, the urine pH of control and test rats were averagely the same for both control and test rats (Table 6), and this explains why animal study results cannot be directly extrapolated to humans.

The histological examination of the sections of the organs revealed that there were no abnormal morphological changes observed in the liver, kidney, heart, lung, and spleen tissues of the treatment groups compared to the control after six weeks of treatment with CSE *C. citratus* ethanolic extract (Figures 4–8). This confirms the results of the biochemical assays which are relatively normal comparing the treatment groups to the control. Saenthaweesuk et al. [44] reported that CSE extracts have a hepatoprotective effect against paracetamol-induced hepatotoxicity in rats. Pretreatment with CSE extracts showed less degree of cellular damage and healing of the hepatocytes following hepatoxicity induction with paracetamol.

### 4.1. Limitations of the Study

The resource equation method used for the sample size calculation is not robust and does not consider the factor of effect size. The current study did not perform a biomarker assessment of cardiotoxicity, lung function, and clotting profile deficiency effect of *C. citratus* CSE ethanolic extract. Moreover, the study did not assess serum pH and bicarbonate to see if there was any acid-base derangement following CSE ethanolic *C. citratus* oral administration. Besides that, urine electrolytes were not measured to assess electrolyte wastage. The study, however, has unraveled the safety profile of *C. citratus* cultivated in Ghana.

## 5. Conclusion

The findings from this study revealed the relative safety of *C. citratus* CSE extracts in Sprague–Dawley rats. However, these findings cannot be extrapolated directly to humans, and people are therefore cautioned to use *C. citratus* infusion or decoction in moderation.

## Figures and Tables

**Table 1:** Acute toxicity study of the effect of conventional Soxhlet *C. citratus* leaves extract in Sprague–Dawley rats over 14 days.

Time	*Mortality*		Toxicity signs
	Dead/treated rat	Latency (h)	
0 hr	0/6	—	None
12 hr	0/6	—	None
24 hr	0/6	—	None
48 hr	0/6	—	None
3 days	0/6	—	None
4 days	0/6	—	None
5 days	0/6	—	None
6 days	0/6	—	None
7 days	0/6	—	None
8 days	0/6	—	None
9 days	0/6	—	None
10 days	0/6	—	None
11 days	0/6	—	None
12 days	0/6	—	None
13 days	0/6	—	None
14 days	0/6	—	None

The ethanolic conventional Soxhlet *C. Citratus* leaves extract was orally administered to six rats at a single dose of 5000 mg/kg bw.t. Observation for signs of toxicity was recorded after (12, 24, and 48) hr and (3, 4, 5, 6, 7, 8, 9, 10, 11, 12, 13, and 14) days after the treatment. The control group was administered with 1 m/100 g bw.t of normal saline. 0/6: no deaths recorded. None: no signs of toxicity were observed over the study period. Latency: time to death (hours) after the treatment. —: latent period.

**Table 2:** Effect of six weeks of oral administration of CSE *C. citratus* ethanolic extract on lipid profile at termination of the experiment.

Parameter	*Treatment group*			
	Control	Conventional Soxhlet ethanolic *C. citratus* extract		
		200 mg/kg	600 mg/kg	1200 mg/kg
Total cholesterol	1.51 ± 0.33	0.52 ± 0.03	0.84 ± 0.35	0.55 ± 0.06
Triglyceride	0.51 ± 0.02	1.81 ± 0.06	2.07 ± 0.26	1.76 ± 0.03
HDL cholesterol	0.98 ± 0.03	0.46 ± 0.04	0.58 ± 0.05	0.35 ± 0.03
LDL cholesterol	0.45 ± 0.07	1.12 ± 0.04	0.92 ± 0.13	1.10 ± 0.03
Coronary risk	1.54 ± 0.32	1.12 ± 0.07	1.46 ± 0.28	1.56 ± 0.06

Values are mean ± SEM of *n* = 6, *p*  >  0.05, compared to control.

**Table 3:** Effect of six weeks of oral administration of CSE *C. citratus* ethanolic extract on haematological parameters at termination.

Parameter	*Treatment group*			
	Control	Conventional soxhlet ethanolic *C. citratus* extract		
		200 mg/kg	600 mg/kg	1200 mg/kg
WBC (×10^9^/L)	18.6 ± 5.21	13.18 ± 4.74	10.15 ± 0.74	10.83 ± 0.74
Lymph# (×10^9^/L)	11.98 ± 2.43	6.63 ± 1.05	6.58 ± 0.64	6.85 ± 0.81
Mid# (×10^9^/L)	1.50 ± 0.35	1.13 ± 0.33	0.88 ± 0.08	1.00 ± 0.14
Gran# (×10^9^/L)	5.13 ± 2.55	5.43 ± 3.39	2.70 ± 0.34	2.98 ± 0.79
Lymph (%)	68.05 ± 4.58	58.73 ± 8.13	64.68 ± 3.65	63.63 ± 6.71
Mid (%)	8.38 ± 0.51	8.80 ± 0.56	8.78 ± 0.42	9.15 ± 1.16
Gran (%)	23.58 ± 4.89	32.48 ± 8.41	26.55 ± 3.48	27.23 ± 5.73
RBC (×10^12^/L)	8.11 ± 0.27	8.43 ± 0.36	8.21 ± 0.15	8.61 ± 0.08
HB (g/dL)	16.70 ± 0.60	15.98 ± 0.39	15.60 ± 0.12	15.88 ± 0.30
HCT (%)	50.48 ± 1.15	48.90 ± 1.40	47.85 ± 0.44	48.05 ± 0.39
MCV (fL)	56.83 ± 1.76	58.18 ± 0.94	58.40 ± 1.55	55.85 ± 0.47
MCH (pg)	18.78 ± 0.71	19.03 ± 0.44	19.00 ± 0.52	18.45 ± 0.31
MCHC (g/dL)	33.03 ± 0.43	32.48 ± 0.37	32.58 ± 0.28	33.08 ± 0.59
RDW-CV (%)	17.63 ± 0.42	18.90 ± 0.29	18.85 ± 0.34	18.18 ± 0.34
RDW-SD (fL)	33.33 ± 1.41	36.55 ± 0.84	36.55 ± 1.02	33.80 ± 0.62
PLT (×10^9^/L)	772.30 ± 60.25	915.25 ± 86.13	912.75 ± 81.81	848.25 ± 33.16
MPV (fL)	6.63 ± 0.23	6.65 ± 0.09	6.70 ± 0.011	6.53 ± 0.08
PDW	14.58 ± 0.09	14.50 ± 0.04	14.55 ± 0.06	14.40 ± 0.04
PCT (%)	0.51 ± 0.04	0.61 ± 0.06	0.61 ± 0.05	0.56 ± 0.02
P-LCR	0.08 ± 0.01	0.07 ± 0.01	0.07 ± 0.01	0.06 ± 0.00

Values are mean ± SEM of *n* = 6, *p*  >  0.05, compared to control.

**Table 4:** Effect of six weeks of oral administration of CSE *C. citratus* ethanolic extract on liver function test at termination of the experiment.

Parameter	*Treatment group*			
	Control	Conventional soxhlet ethanolic *C. citratus* extract		
		200 mg/kg	600 mg/kg	1200 mg/kg
ALT (U/L)	61.08 ± 7.47	79.53 ± 9.33	57.93 ± 4.62	58.45 ± 3.76
AST (U/L)	20.30 ± 5.31	9.95 ± 2.60	22.00 ± 7.90	15.60 ± 0.46
ALP (U/L)	212.83 ± 6.72	294.25 ± 41.82	226.43 ± 40.54	266.78 ± 17.20
GGT (U/L)	7.38 ± 2.42	9.38 ± 4.35	8.00 ± 1.49	4.90 ± 0.68
Total protein (g/L)	79.15 ± 3.09	76.75 ± 2.11	71.40 ± 2.25	73.20 ± 0.88
Albumin (g/L)	36.28 ± 2.09	34.85 ± 1.07	35.78 ± 1.66	34.60 ± 0.50
Total bilirubin (*µ*mol/L)	3.02 ± 0.19	2.73 ± 0.45	3.38 ± 0.32	5.06 ± 1.40
Direct bilirubin (*μ*mol/L)	1.28 ± 0.12	1.14 ± 0.20	1.23 ± 0.23	1.13 ± 0.03
Indirect bilirubin (*μ*mol/L)	2.63 ± 0.78	1.65 ± 0.29	2.15 ± 0.30	2.68 ± 0.66

Values are mean ± SEM of *n* = 6, *p*  >  0.05, compared to control. ALT: alanine aminotransferase; AST: aspartate aminotransferase; ALP: alkaline phosphatase.

**Table 5:** Effect of six weeks of oral administration of CSE *C. citratus* ethanolic extract on renal function test at termination of the experiment.

Parameter	*Treatment group*			
	Control	Conventional soxhlet ethanolic *C. citratus* extract		
		200 mg/kg	600 mg/kg	1200 mg/kg
Urea (mmol/L)	9.12 ± 0.59	8.11 ± 0.27	8.28 ± 0.06	6.79 ± 0.54
Creatinine (*μ*mol/L)	34.53 ± 1.61	30.55 ± 9.75	52.48 ± 13.25	50.28 ± 11.39
Sodium (mmol/L)	142.35 ± 0.85	142.1 ± 1.23	143.15 ± 1.63	142.05 ± 0.37
Chloride (mmol/L)	98.58 ± 1.04	100.13 ± 0.55	99.55 ± 0.45	99.95 ± 0.52
Potassium (mmol/L)	4.73 ± 0.75	5.08 ± 0.41	5.25 ± 0.52	3.75 ± 0.09

Values are mean ± SEM of *n* = 6, *p*  >  0.05, compared to control.

**Table 6:** Effect of six weeks of oral administration of CSE *C. citratus* ethanolic extract on urine parameters at termination.

Parameters	*Treatment group*			
	Control	Conventional soxhlet ethanolic *C. citratus* extract		
		200 mg/kg	600 mg/kg	1200 mg/kg
Glucose (mg/dl)	—	—	—	—
Bilirubin (mg/dl)	—	—	—	—
Ketones (mg/dl)	—	—	—	—
Specific gravity (g/ml)	1.020 ± 0.005	1.016 ± 0.003	1.020 ± 0.000	1.016 ± 0.003
Blood	—	—	—	—
pH	7.38 ± 0.25	7.50 ± 0.50	7.00 ± 0.00	7.62 ± 0.48
Protein (g/L)	—	—	—	—
Nitrite	—	—	—	—
Leukocyte (*μ*l)	—	—	—	—
Urobilinogen (mg/dl)	N	N	N	N

Results presented as means ± SEM of *n* = 6, (—): absent, (N): normal.

## Data Availability

The data used to support the findings of the study are included in the paper.

## References

[1] Manandhar S., Luitel S., Dahal R. K. (2019). In vitro antimicrobial activity of some medicinal plants against human pathogenic bacteria. *Journal of Tropical Medicine*.

[2] Vaou N., Stavropoulou E., Voidarou C., Tsigalou C., Bezirtzoglou E. (2021). Towards advances in medicinal plant antimicrobial activity: a review study on challenges and future perspectives. *Microorganisms*.

[3] Ekor M. (2014). The growing use of herbal medicines: issues relating to adverse reactions and challenges in monitoring safety. *Frontiers in Pharmacology*.

[4] Mensah M. L. K., Komlaga G., Forkuo A. D., Firempong C., Anning A. K., Dickson R. A. (2019). Toxicity and safety implications of herbal medicines used in Africa. *Herbal Medicine*.

[5] Zhang J., Onakpoya I. J., Posadzki P., Eddouks M. (2015). The safety of herbal medicine: from prejudice to evidence. *Evidence-based Complementary and Alternative Medicine*.

[6] Okaiyeto K., Oguntibeju O. O. (2021). African herbal medicines: adverse effects and cytotoxic potentials with different therapeutic applications. *International Journal of Environmental Research and Public Health*.

[7] Aftab K., Ali M. D., Aijaz P. (2011). Determination of different trace and essential element in lemon grass samples by x-ray fluorescence spectroscopy technique. *International Food Research Journal*.

[8] Ekpenyong C. E., Daniel N. E., Antai A. B. (2015a). Bioactive natural constituents from lemongrass tea and erythropoiesis boosting effects: potential use in prevention and treatment of Anemia. *Journal of Medicinal Food*.

[9] Sadi D., Imam T. S. (2019). Effect of Cymbopogon citratus (lemon grass) leaves extract on haematological and biochemical parameters in albino rats (*Rattus norvegicus*). *Katsina Journal of Natural and Applied Sciences*.

[10] Ekpenyong C. E., Daniel N. E., Antai A. B. (2015b). Effect of lemongrass tea consumption on estimated glomerular filtration rate and creatinine clearance rate. *Journal of Renal Nutrition*.

[11] Nogueira A. C. M. A., Carvalho R. R., Souza C. A., Chahoud I., Paumgartten F. J. R. (1995). Study on the embryofeto-toxicity of citral in the rat. *Toxicology*.

[12] Duerksen-Hughes P. J., Yang J., Ozcan O., Duerksen-Hughes P. J. (1999). p53 induction as a genotoxic test for twenty-five chemicals undergoing in vivo carcinogenicity testing. *Environmental Health Perspectives*.

[13] Souza A. C. S., Silva L. K., Queiroz T. B. (2020). Citral presents cytotoxic and genotoxic effects in human cultured cells. *Drug and Chemical Toxicology*.

[14] Plata-Rueda A., Rolim G. D. S., Wilcken C. F., Zanuncio J. C., Serrão J. E., Martínez L. C. (2020). Acute toxicity and sublethal effects of lemongrass essential oil and their components against the granary weevil, Sitophilus granarius. *Insects*.

[15] Ayenew K. D., Sewale Y., Amare Y. E., Ayalew A. (2022). Acute and subacute toxicity study of essential oil of Cymbopogon martini in mice. *Journal of Toxicology*.

[16] Blanco M. M., Costa C. A. R. A., Freire A. O., Santos J. G., Costa M. (2009). Neurobehavioral effect of essential oil of Cymbopogon citratus in mice. *Phytomedicine*.

[17] Costa C. A. R. A., Bidinotto L. T., Takahira R. K., Salvadori D. M. F., Barbisan L. F., Costa M. (2011). Cholesterol reduction and lack of genotoxic or toxic effects in mice after repeated 21-day oral intake of lemongrass (Cymbopogon citratus) essential oil. *Food and Chemical Toxicology*.

[18] Mesa G. M. (2014). Antihypertensive potential of plants used in Cuba. *Pharmacology Online*.

[19] Adeneye A. A., Agbaje E. O. (2007). Hypoglycemic and hypolipidemic effects of fresh leaf aqueous extract of Cymbopogon citratus Stapf. in rats. *Journal of Ethnopharmacology*.

[20] Bimakr M., Rahma R. A., Taip S., Adzahan N. M., Islam S. Z., Ganjloo A. (2013). Ultrasound-assisted extraction of valuable compounds from winter melon (Benincasa hispida) seeds. *International Food Research Journal*.

[21] Mandana B., Russly A. R., Farah S. T., Noranizan M. A., Zaidul I. S., Ali G. (2012). Antioxidant activity of winter melon (Benincasa Hispida) seeds using conventional soxhlet extraction technique. *International Food Research Journal*.

[22] Louhimies S. (2002). Directive 86/609/EEC on the protection of animals used for experimental and other scientific purposes. *Alternatives to Laboratory Animals*.

[23] Wan Mohammad W. M. Z., Zahiruddin W. M. (2017). Sample size calculation in animal studies using resource equation approach. *Malaysian Journal of Medical Sciences*.

[24] Charan J., Kantharia N. (2013). How to calculate sample size in animal studies?. *Journal of Pharmacology and Pharmacotherapeutics*.

[25] Oecd (2001). Test 423: Acute Oral Toxicity-Acute Toxic Class Method. https://www.oecd-ilibrary.org/environment/test-no-423-acute-oral-toxicity-acute-toxic-class-method_9789264071001-en.

[26] Oecd (2018). Test 408: Repeated Dose 90-day Oral Toxicity Study in Rodents. http://www.oecd.org/termsandconditions/.

[27] Donkor K., Antwi S., Asiedu-Larbi J., Takyi N., Okine L. K. (2014a). Sub chronic toxicity studies of Asena, a poly-herbal formulation for the treatment of arthritis in rat. *Medicinal and Aromatic Plant Research Journal*.

[28] Irwin S. (1968). Comprehensive observational assessment: ia. A systematic, quantitative procedure for assessing the behavioral and physiologic state of the mouse. *Psychopharmacologia*.

[29] Oecd (2008). Test 407: Guidelines for the Testing of Chemicals: Repeated Dose 28-day Oral Toxicity Study in Rodents. https://www.oecd-ilibrary.org/.

[30] Farsi E., Shafaei A., Hor S. Y. (2013). Genotoxicity and acute and subchronic toxicity studies of a standardized methanolic extract of ficus deltoidea leaves. *Clinics*.

[31] Kurien B. T., Everds N. E., Scofield R. H. (2004). Experimental animal urine collection: a review. *Laboratory Animals*.

[32] Abotsi W. K. M., Ainooson G. K., Gyasi E. B. (2011). Acute and subacute toxicity studies of the ethanolic extract of the aerial parts of Hilleria Latifolia (Lam.) H. Walt. (Phytolaccaceae) in rodents. *West African Journal of Pharmacy*.

[33] Ahur V. M., Anika S. M., Onyeyili P. A. (2019). Age-sex dimorphisms in the estimation of median lethal dose (LD_50_) of lead diacetate in rabbits using up-and-down procedure (Arithmetic method). *Sokoto Journal of Veterinary Sciences*.

[34] Baig M. W., Majid M., Nasir B. (2022). Toxicity evaluation induced by single and 28-days repeated exposure of withametelin and daturaolone in Sprague Dawley rats. *Frontiers in Pharmacology*.

[35] Baig V. N., Gupta P. K., Sharma A. K., Madhusudan S. (2015). Assessment of knowledge, attitude and practice about hepatitis B among Clinicians & Medical students: a cross sectional study. *National Journal of Community Medicine*.

[36] Nosiri C. I., Nwaogwugwu C. J., Onwuasoanya W. E. (2020). Haematological assessment of ethanolic leaf extract of Cymbopogon citratus in Wistar Rats. *The Pharmaceutical and Chemical Journal*.

[37] Omonije O. O., Saidu A. N., Muhammad H. L. (2019). Anti-diabetic activities of Chromolaena odorata methanol root extract and its attenuation effect on diabetic induced hepatorenal impairments in rats. *Clinical Phytoscience*.

[38] Yusuf A. A., Lawal B., Yusuf M. A. (2018). Free radical scavenging, antimicrobial activities and effect of sub-acute exposure to Nigerian Xylopia aethiopica seed extract on liver and kidney functional indices of albino rat. *Iranian Journal of Toxicology*.

[39] Ramaiah S. K. (2007). A toxicologist guide to the diagnostic interpretation of hepatic biochemical parameters. *Food and Chemical Toxicology*.

[40] Ramaiah S. K. (2011). Preclinical safety assessment: current gaps, challenges, and approaches in identifying translatable biomarkers of drug-induced liver injury. *Clinics in Laboratory Medicine*.

[41] Ahmed S. N., Siddiqi Z. A. (2006). Antiepileptic drugs and liver disease. *Seizure*.

[42] Braide S. A., Davies T. J. (1987). Factors that affect the induction of gamma glutamyltransferase in epileptic patients receiving anti-convulsant drugs. *Annals of Clinical Biochemistry*.

[43] Sarfaraz S., Sarfaraz S., Usmanghani K. (2016). Hepato-protective potential and phytochemical screening of Cymbopogon citratus. *Journal of Analytical & Pharmaceutical Research*.

[44] Saenthaweesuk S., Munkong N., Parklak W., Thaeomor A., Chaisakul J., Somparn N. (2017). Hepatoprotective and antioxidant effects of Cymbopogon citratus Stapf (Lemon grass) extract in paracetamol-induced hepatotoxicity in rats. *Tropical Journal of Pharmaceutical Research*.

[45] Levitt D. G., Levitt M. D. (2016). Human serum albumin homeostasis: a new look at the roles of synthesis, catabolism, renal and gastrointestinal excretion, and the clinical value of serum albumin measurements. *International Journal of General Medicine*.

[46] Chung J. O., Park S.-Y., Chung D. J., Chung M. Y. (2019). Relationship between anemia, serum bilirubin concentrations, and diabetic retinopathy in individuals with type 2 diabetes. *Medicine*.

[47] Tarkang P. A., Agbor G. A., Tsabang N. (2012). Effect of long-term oral administration of the aqueous and ethanol leaf extracts of Cymbopogon citratus (DC. ex Nees) Stapf. *Annals of Biological Research*.

[48] Christopher E. (2018). Lemongrass tea consumption and changes in Acid-Base Balance and Electrolyte homeostasis. *Archive of Food and Nutritional Science*.

